# Effectiveness of systemic treatments for advanced non-clear cell renal cell carcinoma: a systematic review and meta-analysis

**DOI:** 10.3389/fonc.2024.1478245

**Published:** 2024-12-18

**Authors:** Yaping Zhang, Jian Chen, Xiaoyan Wang, Hui Wang, Xiaoli Chen, Jianfeng Hong, Hongming Fang

**Affiliations:** ^1^ Department of Oncology, Affliated Xiaoshan Hospital, Hangzhou Normal University, Hangzhou, China; ^2^ Department of GCP, Affliated Xiaoshan Hospital, Hangzhou Normal University, Hangzhou, China

**Keywords:** non-clear cell renal cell carcinoma, vascular endothelial growth factor receptor tyrosine kinase inhibitors, mammalian target of rapamycin inhibitors, chemotherapy, immune checkpoint inhibitor

## Abstract

**Background:**

Non-clear cell renal cell carcinoma (nccRCC) represents a heterogeneous group of malignancies with substantial differences in morphology, genetic profiles, clinical behavior, and prognosis. Optimal treatment for nccRCC remains unclear, largely extrapolated from evidence available for clear cell renal cell carcinoma (ccRCC). This study aimed to compare the efficacy of current mainstream drug treatments for nccRCC to provide clinical treatment guidance for advanced cases.

**Methods:**

We systematically searched PubMed, Embase, and Cochrane databases for trials published up to January 2, 2024, including controlled and single-arm trials. Primary outcomes included overall response rate (ORR), disease control rate (DCR), progression-free survival (PFS), and overall survival (OS).

**Results:**

We selected six randomized controlled trials (RCTs) comparing mammalian target of rapamycin inhibitors (mTORi) with vascular endothelial growth factor receptor tyrosine kinase inhibitors (VEGFR-TKIs). These trials included four first-line and two second-line studies, with a total of 398 advanced nccRCC patients. Pooled results showed that VEGFR-TKIs significantly improved PFS compared to mTORi in first-line treatment (relative risk [RR] = 1.387; 95% confidence interval [CI]: 1.04-1.85; p = 0.026). In a single-arm meta-analysis, we included 22 VEGFR-TKI trials, three mTORi trials, 12 immune checkpoint inhibitor (ICI) therapies, five chemotherapy trials, and 10 combination therapy trials. The pooled ORR ranged from 6% (95% CI: 0–16%) to 36% (95% CI: 27–44%), and the pooled DCR ranged from 54% (95% CI: 50–58%) to 81% (95% CI: 70–91%). Subgroup analysis of ICI showed a higher ORR in the PD-L1 positive group compared to the PD-L1 negative group (RR = 3.044; 95% CI: 1.623-5.709; p = 0.001).

**Conclusion:**

This systematic review and meta-analysis demonstrate that VEGFR-TKIs improve PFS in first-line treatment compared to mTORi. The single-arm meta-analysis suggest that combination therapies with different mechanisms result in better ORR and DCR. Furthermore, PD-L1 positive patients showed significantly better therapeutic responses with ICI treatment than PD-L1 negative patients.

## 1 Introduction

Renal cell carcinoma (RCC) is a common malignancy of the urinary and reproductive systems, making up 2-3% of systemic malignancies and 80-90% of renal malignancies (1). According to the 2016 World Health Organization (WHO) guidelines, RCC is classified histologically into renal clear cell carcinoma (ccRCC) and non-clear cell carcinoma (nccRCC) (2). NccRCC accounts for 15-30% of renal tumor, and includes various malignancies with significant differences in morphology, genetic profiles, immunohistochemical features, and clinical behaviors (3). Notable subtypes of nccRCC include papillary, chromophobe, collecting duct carcinoma (CDC), renal medullary, spindle cell, mucinous tubular, Xp11.2 translocation, carcinoma associated with neuroblastoma, and other rare entities (2). Papillary RCC (pRCC) and chromophobe RCC (chRCC) are the most prevalent, accounting for 10-15% and 4-5% of renal tumors, respectively (2). The sarcomatoid variant is not a distinct histologic entity but a high-grade transformation seen in various RCC subtypes, characterized by frequent metastasis to the lungs and bones and associated with poor prognosis (4).

Recent advancements in targeted therapy and immunotherapy have significantly improved ccRCC treatment outcomes (5–7). However, due to the heterogeneity and rarity of nccRCC, most RCC clinical trials primarily include ccRCC patients or a small subset of nccRCC patients. As a result, systemic therapy options for nccRCC are largely derived from ccRCC trials and retrospective studies (8, 9). Despite these efforts, nccRCC patients have significantly lower survival rates compared to those with ccRCC (1, 3). The National Comprehensive Cancer Network (NCCN) guidelines recommend clinical trials as the preferred treatment option for nccRCC patients (1), highlighting it as a critical and challenging research area.

Numerous endeavors have focused on developing mesenchymal-epithelial transition (MET) inhibitors, given that MET proto-oncogene has been found to have mutations and copy number changes of pRCC (10). A systematic review and meta-analysis evaluated the efficacy and safety of MET inhibitor (METi) in advanced pRCC, results indicated that the objective response rate (ORR) was 36.38% for MET+ pRCC, and an overall population ORR of 20.56% (11). Subgroup analysis of drugs showed that patients on cabozantinib had an ORR of 26.14%, on savolitinib had an ORR of 15.35% (11). METi represents a promising target for precision therapies, nevertheless, the available data remains limited, requiring further validation of this approach.

Due to the limited clinical trial data and guidance for nccRCC therapy selection, we conducted a systematic review and meta-analysis of existing data. This study aimed to evaluate the antitumor efficacy of vascular endothelial growth factor receptor tyrosine kinase inhibitors (VEGFR-TKIs), mTORi, chemotherapy, immune checkpoint inhibitors (ICIs), and combination therapies in treating nccRCC to provide clinical treatment guidance. Additionally, we analyzed single-arm studies due to the scarcity of controlled trials.

## 2 Methods

### 2.1 Search strategy

This systematic review and meta-analysis evaluated the efficacy of VEGFR-TKIs, mTORi, chemotherapy, ICIs, and combination therapies for advanced nccRCC. Comprehensive searches were performed in the PubMed, Embase, and Cochrane databases for reports published up to January 2, 2024. Only English-language articles and abstracts from all available years were included, while case reports, case series, and review articles were excluded. The primary search term was “renal cell carcinoma”.

### 2.2 Inclusion and exclusion criteria

All trial designs were included, comprising randomized controlled trials (RCTs), non-randomized controlled trials, and single-arm studies.

Inclusion criteria: (1) Adult patients diagnosed histologically or cytologically with advanced nccRCC; (2) Controlled trials or single-arm studies assessing interventions such as VEGFR-TKIs, mTORi, chemotherapy, ICIs, or combination therapies; (3) Studies published in English; (4) Reports of at least one of the following outcomes: ORR, disease control rate (DCR), progression-free survival (PFS), or overall survival (OS); (5) When multiple reports were available from the same investigator for the same patient population, the most recent or comprehensive report was selected.

Exclusion criteria: (1) *In vitro* or *in vivo* studies; (2) Case reports, case series, or studies with fewer than 10 participants; (3) Reviews, abstracts, or letters; (4) Re-challenge therapies; (5) Non-English publications.

### 2.3 Literature screening and data extraction

Two researchers (Y.P.Z. and X.Y.W) independently screened titles and abstracts based on predefined inclusion and exclusion criteria. Data extraction adhered to the Preferred Reporting Items for Systematic Reviews and Meta-Analyses guidelines (12). Discrepancies were resolved through discussion; if consensus was not reached, a third investigator (J.C.) was consulted to arbitrate, with final decisions determined by majority vote. If inclusion could not be established from the abstract or if essential data were missing, the full text was reviewed. A standardized data extraction form was created to collect the following information: study characteristics (first author, publication year, identifier, study design, study phase, treatment type, histological subgroups of nccRCC, treatment line), patient characteristics (number of evaluable patients, number of treatment arms, number of control arms, median age, sex), and outcome assessments (complete response [CR], partial response [PR], stable disease [SD], disease progression [PD], ORR, DCR, PFS, or OS).

### 2.4 Quality assessment

Two investigators (X.L.C. and J.F.H) independently performed the quality assessment. The risk of bias was evaluated in duplicate, with any discrepancies resolved by consensus or by consulting a third reviewer (H.W.). The methodological quality of the included RCTs was assessed using the Cochrane Risk of Bias Tool, as outlined in the Cochrane Handbook for Systematic Reviews of Interventions (13). A risk of bias summary was compiled using Review Manager Version 5.4 (Copenhagen: Nordic Cochrane Centre, Cochrane Collaboration, 2020).

### 2.5 Statistical analysis

Statistical analyses were performed using Stata 12.0 software (Stata Corporation, College Station, TX). Dichotomous data, including ORR and DCR, were compared using relative risk (RR). Hazard ratios (HR) for OS and PFS, along with their 95% confidence intervals (CIs), were extracted from each RCT. Summary HRs were calculated using either random or fixed-effects models, depending on the heterogeneity of the studies. Heterogeneity was assessed using the I² statistic and Chi-squared test, with I² ≥ 50% indicating significant heterogeneity. The random-effects model was employed when heterogeneity was substantial (I² > 50% or P < 0.05); otherwise, the fixed-effects model was applied (14). Additionally, the Begg test and funnel plots were used to assess potential publication bias among the studies. A two-tailed p-value of less than 0.05 was considered statistically significant.

## 3 Results

### 3.1 Literature selection

**Figure 1:**
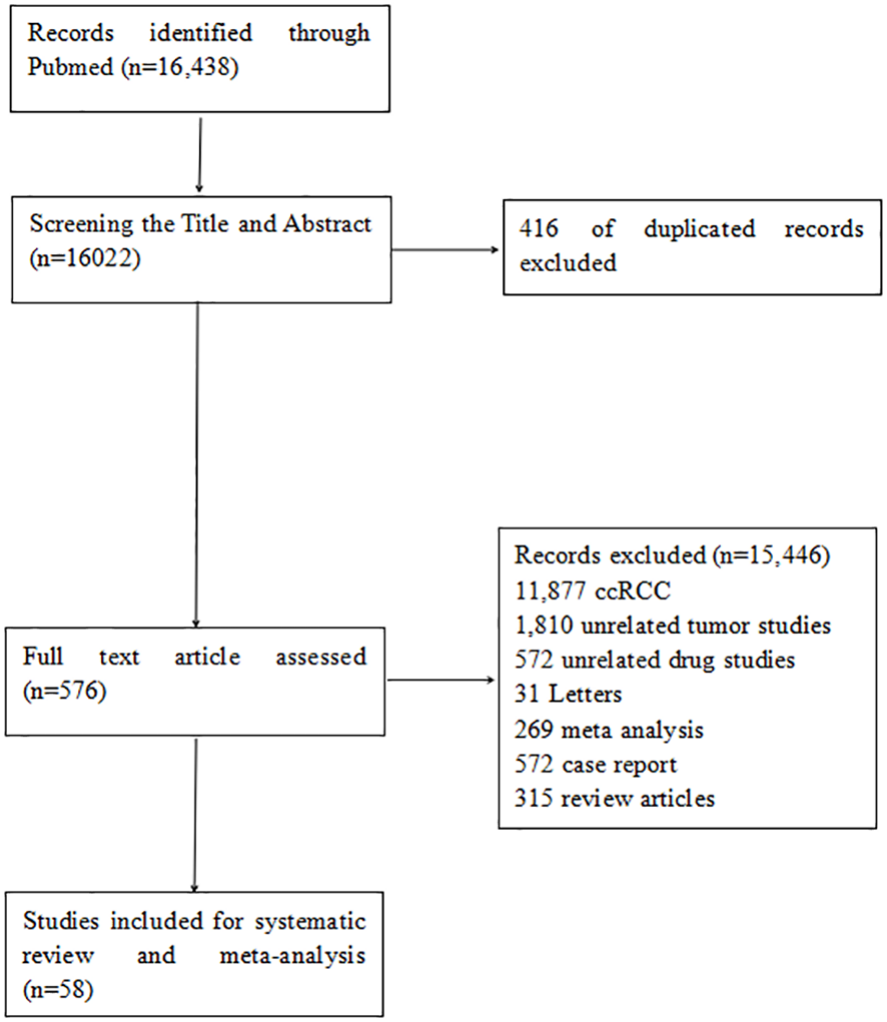
The flowchart of the study selection process for the meta-analysis.

The electronic search identified 16,438 citations published between September 1994 and January 2024. After screening abstracts and titles, 576 full-text articles were reviewed, and 58 studies were included in the systematic review. These comprised six RCTs comparing the efficacy of mTORi and VEGFR-TKIs, 22 single-arm studies on VEGFR-TKIs, three on mTORi, 12 on immunotherapy, five on chemotherapy, and ten on combination therapy. The literature screening process is illustrated in the flowchart in **Figure 1**.

### 3.2 Characteristics of six RCTs comparing mTORi and VEGFR-TKIs

Four studies compared mTORi with VEGFR-TKIs as first-line treatments (15–18), while two studies focused on second-line treatments (15, 19). The ESPN trial provided data for both first-line and second-line treatments separately (15), and we analyzed them as distinct studies. The meta-analysis included 398 patients, with 203 receiving mTORi and 195 receiving VEGFR-TKIs. Among the VEGFR-TKI group, 45 patients were treated with sorafenib, and the rest received sunitinib. Five of the six studies were randomized phase II trials (15–18), and one was a randomized phase III trial (19). Four studies exclusively recruited nccRCC patients (15–17), while two included both ccRCC and nccRCC patients (18, 19), providing separate data for each subgroup. In the three RCTs that reported pathological subgroups (15–17), papillary histology was the most common nccRCC type (113 out of 198 patients). The characteristics and main outcomes of the six RCTs are summarized in **Table 1** and **Table 2**, separately.

**Table 1:** General characteristics of the included randomized controlled trials (RCTs).

First author (year)	Identifier	Study design	Study phase	Patients (n)	Histological subgroups	Comparator	Treatment line
Bergmann (2020)	/	RCT	2	22	Papillary 16 Chromophobe 2 Renal medullary 1 Unclassified 3	Temsirolimus vs Sunitinib	First
Armstrong (2016)	ASPEN	RCT	2	108	Papillary 70 Chromophobe 16 Unclassified 22 Translocation 8 Minor clear cell component 13 Sarcomatoid differentiation 16	Sunitinib vs Everolimus	First
Tannir (2015)	ESPN	RCT	2	68	Papillary 27 Clear cell with sarcomatoid features 12 Chromophobe 12 Translocation 7 Unclassified 10	Everolimus vs Sunitinib	First
Motzer (2014)	RECORD-3	RCT	2	66	/	Everolimus vs Sunitinib	First
Tannir (2015)	ESPN	RCT	2	44	/	Everolimus vs Sunitinib	Second
Hutson (2014)	INTORSECT	RCT	3	90	/	Temsirolimus vs Sorafenib	Second

RCT, randomized controlled trial; n, number.

**Table 2:** Summary of main outcomes of the included RCTs: Response Evaluation Criteria in Solid Tumors (RECIST), progression - free survival (PFS) and overall survival (OS).

First author (year)	Comparator	Age (range)	Sex male/ female	Patients (n)	Response RECIST (n)	PFS (mo)	PFS HR (95% CI)	OS (mo)	OS HR (95% CI)
Bergmann (2020)	Temsirolimus	59.5 (29–85)	8/4	12	CR:0 PR:2 SD:5 PD:2	9.3	1.76 (0.7-4.46)	19.4	0.98 (0.31-3.09)
	Sunitinib	65.5 (46–80)	8/2	10	CR:0 PR:3 SD:6 PD:1	13.2		19.8	
Armstrong (2016)	Everolimus	64 (29–90)	44/13	57	CR:1 PR:4 SD:30 PD:13	5.6	1.41 (0.88-2.26)	13.2	1.12 (0.7-2.1)
	Sunitinib	59 (24–100)	37/14	51	CR:0 PR:9 SD:30 PD:10	8.3		31.5	
Tannir (2015)	Everolimus	58 (23–73)	24/11	35	CR:0 PR:1 SD:26 PD:8	4.1	1.16 (0.67-2.01)	14.9	/
	Sunitinib	60 (28–76)	19/14	33	CR:0 PR:3 SD:21 PD:9	6.1		16.2	
Motzer (2014)	Everolimus	/	/	31	/	5.1	1.5 (0.9-2.8)	/	/
	Sunitinib	/	/	35	/	7.2		/	
Tannir (2015)	Everolimus	/	/	23	PR:2 SD:15 PD:6	2.8	/	/	/
	Sunitinib	/	/	21	SD:13 PD:8	1.8		/	
Hutson (2014)	Temsirolimus	/	/	45	/	/	0.88 (0.53-1.45)		1 .42 (0.86-2.35)
	Sorafenib	/	/	45	/	/			

HR, hazard ratio; CI, confidence interval; n, number; CR, complete response; PR, partial response; PD, progressive disease; SD, stable disease.

### 3.3 Assessment of study quality and risk of bias

**Figure 2:**
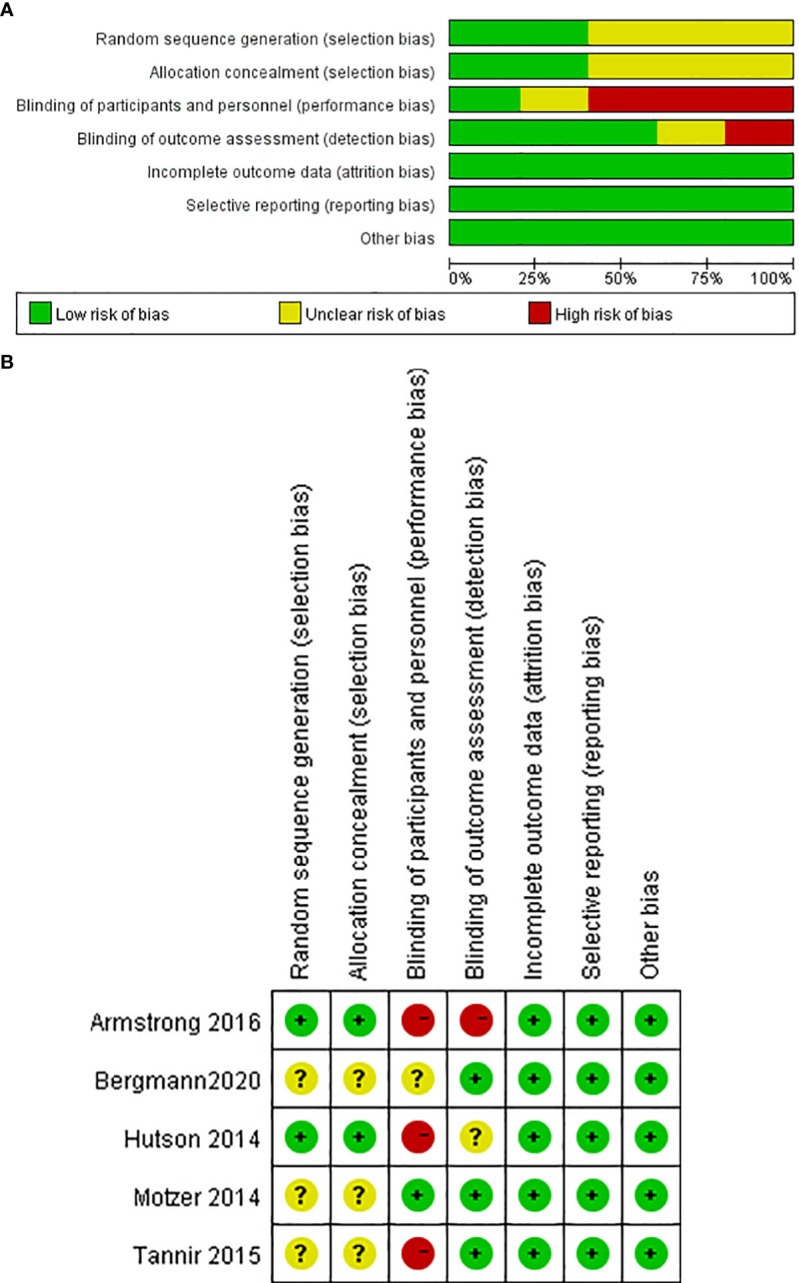
Risk of bias. **(A)** Graph: review authors’ judgments about each risk of bias item presented as percentages across all RCTs. **(B)** Summary: review authors’ judgments about each risk of bias item for each included RCT.

A total of 398 patients were included in the six RCTs. Five studies reported PFS (15–19), four reported ORR and DCR (15–17), and three reported OS (16, 17, 19). The studies exhibited some risks of bias, primarily in blinding of participants and personnel (15–17, 19), random sequence generation (15, 17, 19) and allocation concealment (15, 17, 19). **Figure 2** presents the risk of bias summary and graph.

### 3.4 PFS and OS

**Figure 3:**
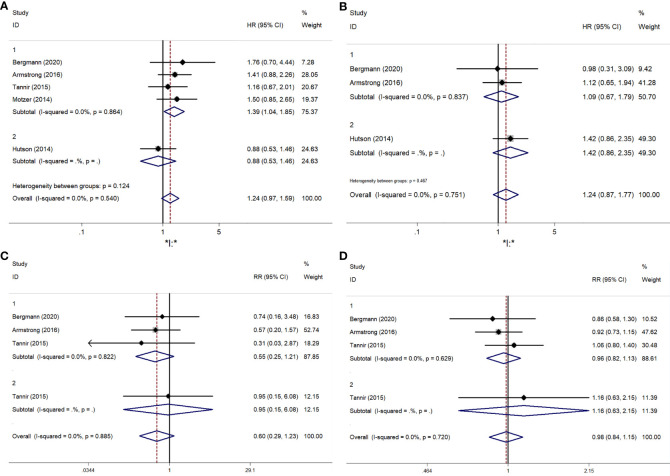
The forest plot comparing of PFS, OS, ORR, DCR between mTORi and VEGFR-TKIs. **(A)** PFS, **(B)** OS, **(C)** ORR, **(D)** DCR.

A comparative meta-analysis of six RCTs examined the PFS of mTORi versus VEGFR-TKIs. Four of these trials were first-line treatments (15–18). The overall analysis showed no significant difference in PFS between the two treatment groups (RR = 1.240; 95% CI, 0.966-1.592; P = 0.091). However, a subgroup analysis revealed a significant PFS benefit for sunitinib over mTORi in first-line treatment (RR = 1.387; 95% CI, 1.040-1.850; P = 0.026) (**Figure 3A**). There was no significant heterogeneity among the studies.

Three articles provided specific OS data: two compared mTORi to VEGFR-TKIs in first-line treatment (16, 17), and one in second-line treatment (19). The results showed no significant difference in OS between mTORi and VEGFR-TKIs (RR = 1.243; 95% CI, 0.874-1.769; P = 0.227) (**Figure 3B**). Similar results were observed in the subgroup analysis of first- and second-line treatments.

#### 3.4.1 ORR and DCR

The meta-analysis results for ORR and DCR are shown in **Figures 3C**, **D**. Statistical tests indicated low heterogeneity for both ORR (I² = 0%, P = 0.885) and DCR (I² = 0%, P = 0.720). Sunitinib did not demonstrate a significant advantage over mTORi in ORR (RR = 0.597; 95% CI, 0.289-1.232; P = 0.163) or DCR (RR = 0.983; 95% CI, 0.837-1.153; P = 0.830). Subgroup analysis for first-line and second-line treatments also showed no significant differences in ORR (RR = 0.548; 95% CI, 0.248-1.209; P = 0.136) or DCR (RR = 0.960; 95% CI, 0.817-1.127; P = 0.616).

### 3.5 Single-arm trials with VEGFR-TKIs

#### 3.5.1 Characteristics

All included studies evaluated the efficacy of VEGFR-TKIs for advanced nccRCC. Among the 22 clinical trials, 12 were prospective (20–31) and 10 were retrospective (32–41), comprising a total of 1,597 patients. Of the 18 studies that specified pathological subgroups (21, 24–39, 41), papillary histology was the most common, accounting for 607 of 914 patients. The most frequently used VEGFR-TKI was sunitinib (1,025 patients), followed by sorafenib (192 patients), pazopanib (111 patients), axitinib (58 patients), and tivozanib (46 patients). Five studies involved only first-line treatments (20, 21, 32–34), while the remaining 17 either included patients with prior anti-tumor treatments or did not specify treatment history. Detailed information on these 22 single-arm experiments is provided in **Table 3** and **Table 4**.

**Table 3:** General characteristics of the included single-arm trials.

Treatment group	First author	Identifier	Study design	Study phase	Patients (n)	Histological subgroups	Treatment	Treatment line
VEGFR-TKIs	Ravaud (2015)	SUPAP	Prospective	2	61	Papillary type I 15 Papillary type II 46	Sunitinib	First
	Procopio (2022)	Meet-URO 2/ NCT03354884	Prospective	2	23	Collecting duct 23	Cabozantinib	First
	Gore (2009) ^a^	NCT00130897	Prospective	/	588	/	Sunitinib	Unselected
	Kim (2011) ^a^	/	Prospective	/	18	/	Sunitinib	Unselected
	Barata (2023)	/	Prospective Randomized	2	46	Papillary 11 Chromophobe 2 Collecting duct 2 Mixed/unclassified 31	Tivozanib	First Second
	Tannir (2012)	NCT00465179	Prospective	2	57	Papillary 27 Chromophobe 5 Collecting duct or medullary carcinoma 6 Sarcomatoid 7 Unclassified 8 Others 4	Sunitinib	First Second Third
	Molina (2012)	/	Prospective	2	23	Papillary 8 Chromophobe 2 Collecting duct 4 Medullary 1 Unclassified 5 HLRCC-related 3	Sunitinib	Unselected
	Lee (2012)	/	Prospective	2	31	Papillary 22 Chromophobe 3 Unclassified 5 Xp11.2 translocation type 1	Sunitinib	Unselected
	Jung (2018)	/	Prospective Multicenter	2	26	Papillary 19 Chromophobe 3 Unclassified 2 Unknown 5	Pazopanib	Unselected
	Park (2018)	/	Prospective Multicenter	2	40	Papillary 26 Chromophobe 4 MiT family translocation 7 Others 3	Axitinib	Unselected
	Procopio (2007)	/	Prospective	/	23	Papillary 14 Chromophobe 3 Bellini ducts 1 Sarcomatoid variants 1 Mixed or unknown 4	Sorafenib	Unselected
	Stadler (2010)	NCT00111020	Prospective	/	127	Papillary 107 Chromophobe 20	Sorafenib	Unselected
	Poprach (2019) ^a^	/	Retrospective	/	93	Papillary 87 Chromophobe 6	Sunitinib or pazopanib	First
	Buti (2021)	/	Retrospective	/	48	Papillary 24 Chromophobe 9 Xp11 translocation 1 Unclassified 6 Mixed 8	Pazopanib	First
	Koguchi (2023) ^a b^	/	Retrospective	/	60	/	Sunitinib or sorafenib or axitinib or pazopanib	First
	SHI (2015)	/	Retrospective	/	37	Papillary 25 Chromophobe 2 Spindle cell type 2 Unclassified 8	Sunitinib	Unselected
	Paglino (2012)	/	Retrospective	/	21	Papillary type I 4 Papillary type II 2 Chromophobe 4 Tubular, mucinous and spindle cell 1 Bellini’s collecting duct carcinoma 4 Mixed 1 Sarcomatoid component ≥20% 5	Sunitinib	Unselected
	YILDIZ (2014)	/	Retrospective	/	63	Papillary 46 Chromophobe 10 Undifferentiated 7	Sunitinib	Unselected
	Campbell (2018)	/	Retrospective	/	30	Papillary 17 Chromophobe 6 Unclassified 3 Translocation 2 Sarcomatoid 1 Mucinous tubular/spindle cell 1	Cabozantinib	Unselected
	Chanzá (2019)	/	Retrospective	/	112	Papillary 66 Xp11-2 translocation 17 Unclassifid 15 Chromophobe 10 Collecting duct 4	Cabozantinib	Unselected
	Matrana (2016) ^c^	/	Retrospective	/	17	/	Pazopanib	Not first-line
	Choueiri (2008)	/	Retrospective	/	53	Papillary 41 Chromophobe 12	Sunitinib or sorafenib	Unselected
mTORi	Escudier (2016)	RAPTOR	Prospective	2	88	Papillary 88	Everolimus	First
	Koh (2013)	NCT00830895	Prospective	2	49	Papillary 29 Chromophobe 8 Collecting duct 2 Sarcomatoid 4 Unclassifiable 6	Everolimus	Unselected
	Lee (2019)	/	Prospective Retrospective		44	Papillary 24 Chromophobe 11 Collecting duct 2 Xp11.2 translocation 1 Others 6	Temsirolimus	Unselected
ICIs	Vogelzang (2020) ^a^	CheckMate 374	Prospective	3b/4	44	/	Nivolumab	Unselected
	Atkins (2023) ^d^	NCT03117309	Prospective	2	35	Papillary 19 Chromophobe 6 Unclassified 10	Nnivolumab	First
	Albiges (2020)	NCT03012581	Prospective	2	50	Papillary type I 5 Papillary type II 20 Chromophobe 9 pRCC unclassified 4 Collecting duct 4 Others 8	Nivolumab	First Second Third
	Conduit (2023) ^d^	NCT03177239	Prospective	2	83	Papillary 37 Chromophobe 15 Other 31	Ipilimumab	Unselected (no prior ICIs)
	McDermott (2021)	KEYNOTE-427 study (cohort B)	Prospective	2	165	Papillary 118 Chromophobe 21 Unclassifified 26	Pembrolizumab	First
	Tykodi (2022)	CheckMate 920 (NCT02982954)	Prospective	3b/4	52	Papillary 18 Chromophobe 7 Translocation-associated 2 Collecting duct 2 Renal medullary 1 Unclassified 22	Ipilimumab plus nivolumab	First
	Koguchi (2023) ^b^	/	Retrospective	/	16	/	Nivolumab	Second
	Koshkin (2018)	/	Retrospective	/	41	Papillary 16 Unclassified 14 Chromophobe 5 Collecting duct 4 Ranslocation 1 Mucinous Tubular and spindle cell carcinoma 1	Nivolumab	Unselected
	CHAHOUD (2019)	/	Retrospective	/	40	Papillary 12 Chromophobe 5 Unclassified 11 Other 12	Nivolumab	Unselected
	Vries-Brilland (2020)	/	Retrospective	/	57	Papillary type I 16 Papillary type II 34 Papillary unclassified 7	PD-1/PD-L1 inhibitors	Unselected
	McKay (2018)	/	Retrospective	/	43	Papillary 14 Chromophobe 10 Unclassified 9 Other 10	PD-1/PD-L1 inhibitors	Unselected
	Gupta (2019)	/	Retrospective	/	18	Papillary 6 Chromophobe 5 Unclassified 3 Other 4	Ipilimumab +nivolumab	Unselected
Chemotherapy	Tsimafeyeu (2012)	NCT01182142	Prospective	2	51	Papillary 39 Chromophobe 7 Collecting duct 5	Capecitabine	Unselected
	Oudard (2007)	GETUG	Prospective	2	23	Collecting duct 23	Gemcitabin plus platinum salt	First
	Bylow (2009)	/	Prospective	2	17	Collecting duct 1 Papillary 16	Carboplatin plus paclitaxel	First
	Richey (2013)	/	Prospective	2	15	Papillary 5 Collecting duct 3 Chromophobe 2 Unclassified 3 Translocation carcinoma 2	Pemetrexed plus gemcitabine	Unselected (no prior chemotherapy)
	Rizzo (2022)	/	Retrospective	/	36	Collecting duct 36	Cisplatin-based chemotherapy	First
Combination therapy	Hutson (2021)	/	Prospective	2	31	Papillary 20 Chromophobe 9 Unclassified 2	Lenvatinib plus everolimus	First
	Feldman (2020)	/	Prospective	2	39	Unclassified RCC, Papillary features 24 Papillary 14 Translocation-associated RCC, papillary features 1	Everolimus plus bevacizumab	First
	Albiges (2023)	KEYNOTE-B61	Prospective	2	158	Papillary 93 Chromophobe 29 Unclassified 21 Translocation 6 Other 9	Pembrolizumab plus lenvatinib	First
	Mahoney (2016) ^a^	/	Prospective	2	13	Papillary 6 Unclassifified 5 Chromophobe 1 Poorly differentiated RCC which was included as nccRCC 1	Bevacizumab plus temsirolimus	Not first-line
	McGregor (2019)	/	Prospective	2	60	Papillary 12 Chromophobe10 Collecting duct 5 Medullary 1 Translocation 5 Unclassified RCC with or without sarcomatoid differentiation 9 ccRCC With 20% sarcomatoid differentiation 18	Atezolizumab plus bevacizumab	Unselected
	Suarez (2023)	NCT02819596	Prospective	2	41	Papillary 41	Savolitinib plus durvalumab	Unselected
	Sheng (2018)	NCT01762150	Prospective	2	26	collecting duct carcinoma 28	Sorafenib in combination with gemcitabine plus cisplatin	Unselected (no prior chemotherapy)
	Srinivasan (2023)	NCT01130519	Prospective	2	83	HLRCC 42 Sporadic 41	Bevacizumab plus erlotinib	Unselected
	Lee (2022)	NCT03635892	Prospective	2	40	Papillary 32 Unclassified without papillary features 6 Translocation-associated 2	Cabozantinib plus nivolumab	First Second
	Stellato (2023)	Meet-URO 23a	Prospective retrospective	/	32	Chromophobe 13 Papillary 19	Pembrolizumab plus axitinib	First

VEGFR-TKIs, vascular endothelial growth factor receptor tyrosine kinase inhibitors; mTORi, mammalian target of rapamycin inhibitors; ICI, immune checkpoint inhibitors; n, number.

a Study includes ccRCC and nccRCC, with some data extracted.

b Two sets of data from the same study.

c This study includes 9 cases of first-line treatment, but not included because of less than 10 cases.

d This study includes combination of nivolumab/ipilimumab treatment after immunotherapy for PD, but was not included.

**Table 4:** Summary of main outcomes of the included studies: Response Evaluation Criteria in Solid Tumors (RECIST), progression-free survival (PFS) and overall survival (OS).

Treatment group	First author	Treatment	Age (Range)	Sex male/ female	Patients (n)	Patients for response/ survival analysis (n)	Response RECIST (n)	PFS (mo) (95% CI)	OS (mo) (95% CI)
VEGFR-TKIs	Ravaud (2015)	Sunitinib	64 (32-81)	51/10	61	60/60	CR: 0 PR: 7 SD: 35 PD: 18	6.6 (2.8-14.8)	17.8 (5.7-26.1)
	Procopio (2022)	Cabozantinib	66 (53-74)	19/4	23	23/23	CR: 1 PR: 7 SD: 3	4 (3-13)	7 (3-31)
	Gore (2009)	Sunitinib	/	/	588	437/588	CR: 2 PR: 46 SD: 250 PD: 139	7.8 (6.3–8.3)	13.4 (10.7–14.9)
	Kim (2011)	Sunitinib	/	/	18	18/18	CR: 0 PR: 3 SD: 13 PD: 2	5.8 (4.0-7.6)	/
	Barata (2023)	Tivozanib	55 (26-75)	34/12	46	46/46	PR: 7 SD: 22 PD: 12 NE: 5	6.7 (3.9-12)	/
	Tannir (2012)	Sunitinib	57 (22-85)	38/19	57	55/55	CR: 0 PR: 3 SD: 29 PD: 23	2.7 (1.4-5.4)	16.8 (10.7-26.3)
	Molina (2012)	Sunitinib	55 (21-80)	17/6	23	23/23	PR: 1 SD: 15 PD: 6 NE: 1	5.5 (2.5-7.1)	/
	Lee (2012)	Sunitinib	53 (18-76)	23/8	31	31/31	PR: 11 SD: 17 PD: 1 NE: 2	6.4 (4.2-8.6)	/
	Jung (2018)	Pazopanib	58 (27-76)	21/8	29	28/28	CR: 0 PR: 8 SD: 17 PD: 3	16.5 (10.9-22.1)	/
	Park (2018)	Axitinib	59 (22-84)	26/14	40	40/40	PR: 15 SD: 12 PD: 11 NE: 2	7.4 (5.2-9.5)	12.1 (6.4-17.7)
	Procopio (2007)	Sorafenib	/	/	23	23/23	CR: 0 PR: 1 SD: 4 PD: 18	/	/
	Stadler (2010)	Sorafenib	/	/	127	127/127	CR: 0 PR: 4 SD: 104 PD: 19	/	/
	Poprach (2019)	Sunitinib or pazopanib	63 (33-82)	68/25	93	83/93	CR: 1 PR: 11 SD: 28 PD: 31 NE: 12	6.5 (2.5−10.5)	22 (14.6−29.4)
	Buti (2021)	Pazopanib	70 (27-86)	36/12	48	48/48	CR: 0 PR: 13 SD: 27 PD: 6 NE: 2	12.3 (3.6-20.9)	27.7 (18.2-37.1)
	Koguchi (2023)	Sunitinib or sorafenib or axitinib or pazopanib	63 (24–89)	38/22	60		/	5.4 ( 2.5-7.1)	/
	SHI (2015)	Sunitinib	50 (29–74)	26/11	37	37/37	CR: 0 PR: 5 SD: 22 PD: 10	6 (3.6−8.4)	9 (6.9−11.1)
	Paglino (2012)	Sunitinib	54.9 (35–74)	20/1	21	21/21	CR: 1 PR: 2 SD: 8 PD: 10	4.08 (average:10.60; range: 1.38-73.1) ^e^	14.60 (average: 27.62;range:2.04-78.1) ^e^
	YILDIZ (2014)	Sunitinib	63 (25–82)	38/25	63	63/63	CR: 0 PR: 7 SD: 33 PD: 23	7.6 (5.5-9.7)	22 (13.4-30.6)
	Campbell (2018)	Cabozantinib	58.4 (25-81)	26/4	30	28/30	PR: 4 SD: 18 PD: 6	8.6 (6.1-14.7)	25.4 (15.3-35.4)
	Chanzá (2019)	Cabozantinib	60 (48–66)	85/27	112	112/112	CR: 1 PR: 29 SD: 53 PD: 25 NE: 4	7.0 (5.7–9.0)	12.0 (9.2–17.0)
	Matrana (2016)	Pazopanib	/	/	17	17/17	PR: 1 SD: 14 PD: 2	4.0 (2.1–9.9)	13.6 (6.4–NA)
	Choueiri (2008)	Sunitinib or sorafenib	59 (24-83)	34/19	53	53/53	CR: 0 PR: 5 SD: 36	8.6 (5.3-11.9)	/
mTORi	Escudier (2016)	Everolimus	60 (23-84) ^f^	72/20 ^f^	88	88/88	CR: 0 PR: 1 SD: 57 PD: 28 Unknown: 2	4.1 (3.6-5.5)	21.4 (15.4-28.4)
	Koh (2013)	Everolimus	57.0 (23.8-75.5)	37/12	49	49/49	PR: 5 SD: 25 PD: 16 Unable: 3	5.2 ^e^	14.0 ^e^
	Lee (2019)	Temsirolimus	52 (17-84)	32/12	44	35/44	CR: 3 PR: 1 SD: 25 PD: 6	7.6 (5.0-10.2)	17.6 (0-39.1)
ICIs	Vogelzang (2020)	Nivolumab	62.0 (32-89)	32/12	44	44/44	CR: 1 PR: 5 SD: 16 PD: 18 Unable to determine: 4	2.2 (1.8-5.4)	16.3 (9.2-NE)
	Atkins (2023)	Nivolumab	63 (35–84)	31/4	35	35/35	CR: 2 PR: 3 SD: 16 PD: 14	4.0 (2.7-4.3)	/
	Albiges (2020)	Nivolumab	61.4	35/15	50	50/50	CR+PR: 5 SD+PD: 45	3.9 (2.9-8.3)	/
	Conduit (2023)	Ipilimumab	64 (21–88)	57/26	83	83/83	CR: 3 PR: 11 SD: 39 PD: 27 Unknown: 3	4.0 (3.6-7.4)	24 (16–28)
	McDermott (2021)	Pembrolizumab	62 (22-86)	109/56	165	165/165	CR: 11 PR: 33 SD: 51 PD: 60 NE: 2 NA: 8	4.2 (2.9-5.6)	28.9 (24.3–NR)
	Tykodi (2022)	Ipilimumab +nivolumab	64 (23–86)	36/16	52	46/52	CR: 2 PR: 7 SD: 17 PD: 19 Unable to determine: 1	3.7 (2.7-4.6)	21.2 (16.6-NE)
	Koguchi (2023)	Nivolumab	64 (30–73)	11/5	16	/	/	5.6 (2.4-10.3)	
	Koshkin (2018)	Nivolumab	58 (33–82)	29/12	41	41/41	PR: 7 SD: 10 PD: 18 NE: 6	3.5 (1.9–5.0)	NR
	CHAHOUD (2019)	Nivolumab	/	/	31	31/31	CR: 2 PR: 1 SD: 13 PD: 9 NA: 6	4.3 (3.4-7)	11.6 (6.1-22.8)
	Vries-Brilland (2020)	PD-1/PD-L1 inhibitors	65 (19-85)	43/14	57	57/57	CR: 2 PR: 4 SD: 18 PD: 31 Unknown: 2	3.1 (2.7-5.0)	14.6 (9.0-NA)
	McKay (2018)	PD-1/PD-L1 inhibitors	/	/	43	30/43	CR: 0 PR: 4 SD: 11 PD: 15	4.6 (2.8-6.0)	/
	Gupta (2019)	Ipilimumab +nivolumab	60 (32-81)	14/4	18	18/18	PR: 6 SD: 3 PD: 9	7.1 ^e^	/
Chemotherapy	Tsimafeyeu (2012)	Capecitabine	/	37/14	51	51/51	CR: 2 PR: 11 SD: 24 PD: 14	10.1 (8.7-11.5)	18.3 (15.5-21.1)
	Oudard (2007)	Gemcitabine+platinum Salt	65 (18–74)	10/13	23	23/23	CR: 1 PR: 5 SD: 10 PD: 7	7.1 (3-11.3)	10.5 (3.8-17.1)
	Bylow (2009)	Carboplatin +paclitaxel	55 (36-67)	15/2	17	17/17	CR: 1 PR: 0 SD: 8 PD: 4 NE: 4	/	/
	Richey (2013)	Pemetrexed +gemcitabine	58.5 (18-77)	12/3	15	14/14	CR: 0 PR: 0	3.2 (1.9-6+)	23.2 (12.9-38.1)
	Rizzo (2022)	Cisplatin-based chemotherapy	66.6(51-77)	26/10	36	36/36	CR: 0 PR: 8 SD: 9 PD: 19	6 (4.95-6.8)	8 (7.4-9.8)
Combination therapy	Hutson (2021)	Lenvatinib plus everolimus	64 (38–85)	20/11	31	31/31	CR: 0 PR: 8 SD: 18 PD: 3 NE/unknown: 2	9.2 (5.5–NE)	15.6 (9.2–NE)
	Feldman (2020)	Everolimus plus bevacizumab	54 (27-77)	32/7	39	37/39	CR: 0 PR: 13 SD: 21 PD: 3	13.7 (10.8-16.4)	33.9 (23.3-71.9)
	Albiges (2023)	Pembrolizumab plus lenvatinib	60 (52-69)	112/46	158	158/158	CR: 9 PR: 69 SD: 52 PD: 17 NE: 1 NA: 10	18 (14–NR)	NR
	Mahoney (2016)	Bevacizumab plus temsirolimus	/	/	13	13/13	PR: 1 SD: 10 PD: 2	5.6 (3.4-13.7)	13.1 (5.0-24.6)
	McGregor (2019)	Atezolizumab plus bevacizumab	61 (22-82)	47/13	60	60/60	CR+PR: 20 SD+PD: 40 ^g^	8.3 (5.7-10.9)	NR
	Suarez (2023)	Savolitinib plus durvalumab	62 (22-77)	34/7	41	41/41	CR: 0 PR: 11 SD: 4 PD: 20 NE: 4 NA: 2	4.9 (2.5-10.0)	14.1 (7.3-30.7)
	Sheng (2018)	Sorafenib in combination with gemcitabine plus cisplatin	/	21/5	26	26/26	CR: 0 PR: 8 SD: 14 PD: 4	8.8 (6.7-10.9)	12.5 (9.6-15.4)
	Srinivasan (2023)	Bevacizumab plus erlotinib	/	/	83	83/83	CR+PR: 42	14.2 (11.4-18.6)	/
	Lee (2022)	Cabozantinib plus nivolumab	57 (33-78)	28/12	40	40/40	PR: 19 SD: 20 PD: 1	12.5 (6.3-16.4)	28 (16.3-NE)
	Stellato (2023)	Pembrolizumab plus axitinib	68	23/9	32	32/32	CR: 0 PR: 14 SD: 11 PD: 6 NE: 1	10.8 (7.8–13.7)	NR

VEGFR-TKIs, vascular endothelial growth factor receptor tyrosine kinase inhibitors; mTORi, mammalian target of rapamycin inhibitors; ICI, immune checkpoint inhibitors; n, number; CI, confidence interval; CR, complete response; PR, partial response; SD, stable disease; PD, progressive disease; NA, not available; NE, not estimable; NR, not reached.

e The 95% CI for PFS and OS was not provided.

f The data is sourced from the safety population (N=92) mentioned in the article.

g The literature indicates that the ORR was 33% (n=20)

#### 3.5.2 ORR and DCR

**Figure 4:**
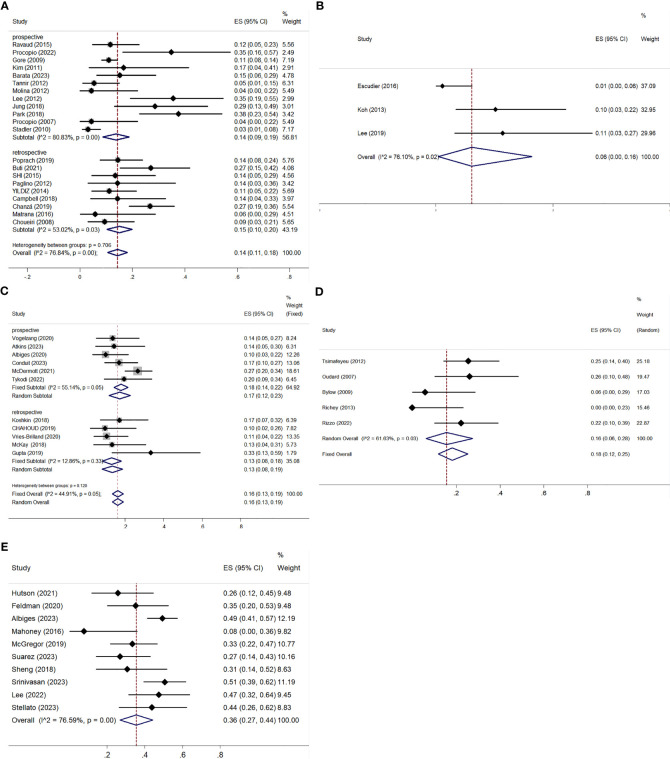
ORR for patients with nccRCC receiving VEGFR-TKIs, mTORi, ICIs, chemotherapy, and combination therapy. **(A)** VEGFR-TKIs, **(B)** mTORi, **(C)** ICIs, **(D)** chemotherapy, **(E)** combination therapy.

**Figure 5:**
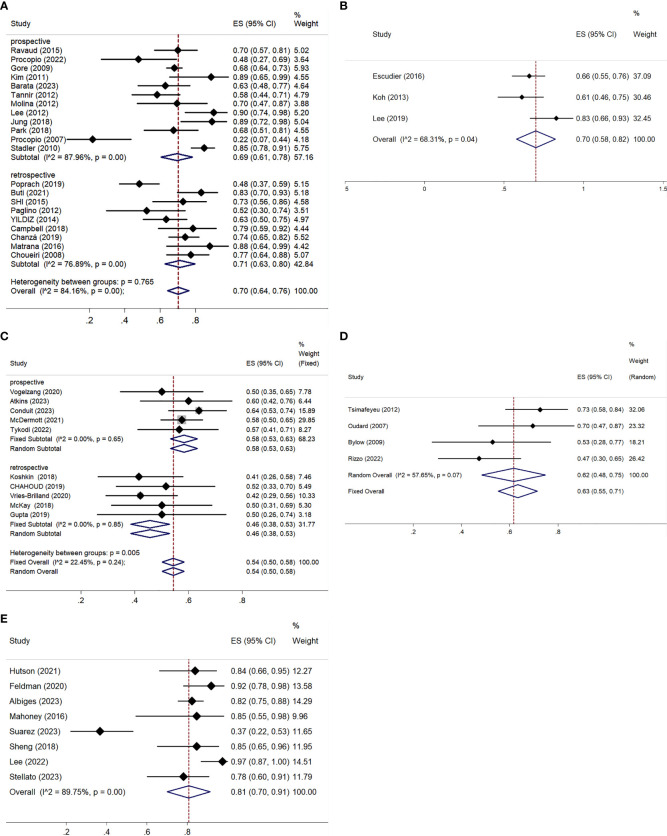
DCR for patients with nccRCC receiving VEGFR-TKIs, mTORi, ICIs, chemotherapy, and combination therapy. **(A)** VEGFR-TKIs, **(B)** mTORi, **(C)** ICIs, **(D)** chemotherapy, **(E)** combination therapy.

All studies, except one retrospective trial (32), provided analyzable data for ORR and DCR. Both ORR and DCR exhibited significant heterogeneity, with I² values over 50% and p-values below 0.01 in the Q-test, indicating notable variability among the studies. The ORR ranged from 3.15% to 35.48%, with a pooled ORR of 14% (95% CI: 11–18%) (**Figure 4A**). Subgroup analysis showed a pooled ORR of 14% (95% CI: 9–19%) in prospective studies and 15% (95% CI: 10–20%) in retrospective studies. The DCR varied from 21.74% to 90.21%, with a pooled DCR of 70% (95% CI: 64–76%) (**Figure 5A**). Subgroup analysis revealed a pooled DCR of 69% (95% CI: 61–78%) in prospective studies and 71% (95% CI: 63–80%) in retrospective studies.

Sensitivity analysis for both ORR and DCR showed no significant interference from any single study, confirming the reliability of the meta-analysis results.

### 3.6 Single-arm trials with mTORi

#### 3.6.1 Characteristics

Three relevant studies were included in this analysis after screening: RAPTOR (42), NCT00830895 (43), and Lee (44). RAPTOR (42) and NCT00830895 (43) were open-label, single-arm, non-randomized, multicenter studies, while Lee (16) integrated both prospective and retrospective data. A total of 215 patients were involved, with 181 in prospective analyses and 34 in retrospective studies. All 88 patients in RAPTOR (42) had pRCC. In the other two studies, pRCC was also the most common, followed by chRCC. Two studies used everolimus, while the third used temsirolimus. Only RAPTOR (42) included patients who had not received previous systemic therapy. Detailed information on these three single-arm studies is presented in **Table 3** and **Table 4**.

#### 3.6.2 ORR and DCR

The three articles provided data on ORR and DCR (42–44). The pooled ORR was 6% (95% CI: 0–16%), with significant heterogeneity (I² = 76.10%, P = 0.02) (**Figure 4B**). The pooled DCR was 70% (95% CI: 58–82%), also with high heterogeneity (I² = 68.31%, P = 0.04) (**Figure 5B**).

### 3.7 Single-arm trials with ICIs

#### 3.7.1 Characteristics

A total of 622 patients participated in six prospective (45–50) and six retrospective studies (32, 51–55). Prior treatment with ICIs was excluded. Only three studies received first-line treatment (46–48). Among the prospective studies, two were Phase IIIb/IV trials (45, 48). Two retrospective studies analyzed various PD-1/PD-L1 inhibitors (53, 54). Nivolumab monotherapy was the most commonly used, appearing in six studies (32, 45, 46, 49, 51, 52). Combination therapy with ipilimumab and nivolumab was examined in two studies (48, 55). Both Vogelzang (45) and Koguchi (32) researched ccRCC and nccRCC but did not detail the nccRCC subtypes. Patients with pRCC were the most prevalent. Detailed information on these studies is presented in **Table 3** and **Table 4**.

#### 3.7.2 ORR and DCR

**Figure 6:**
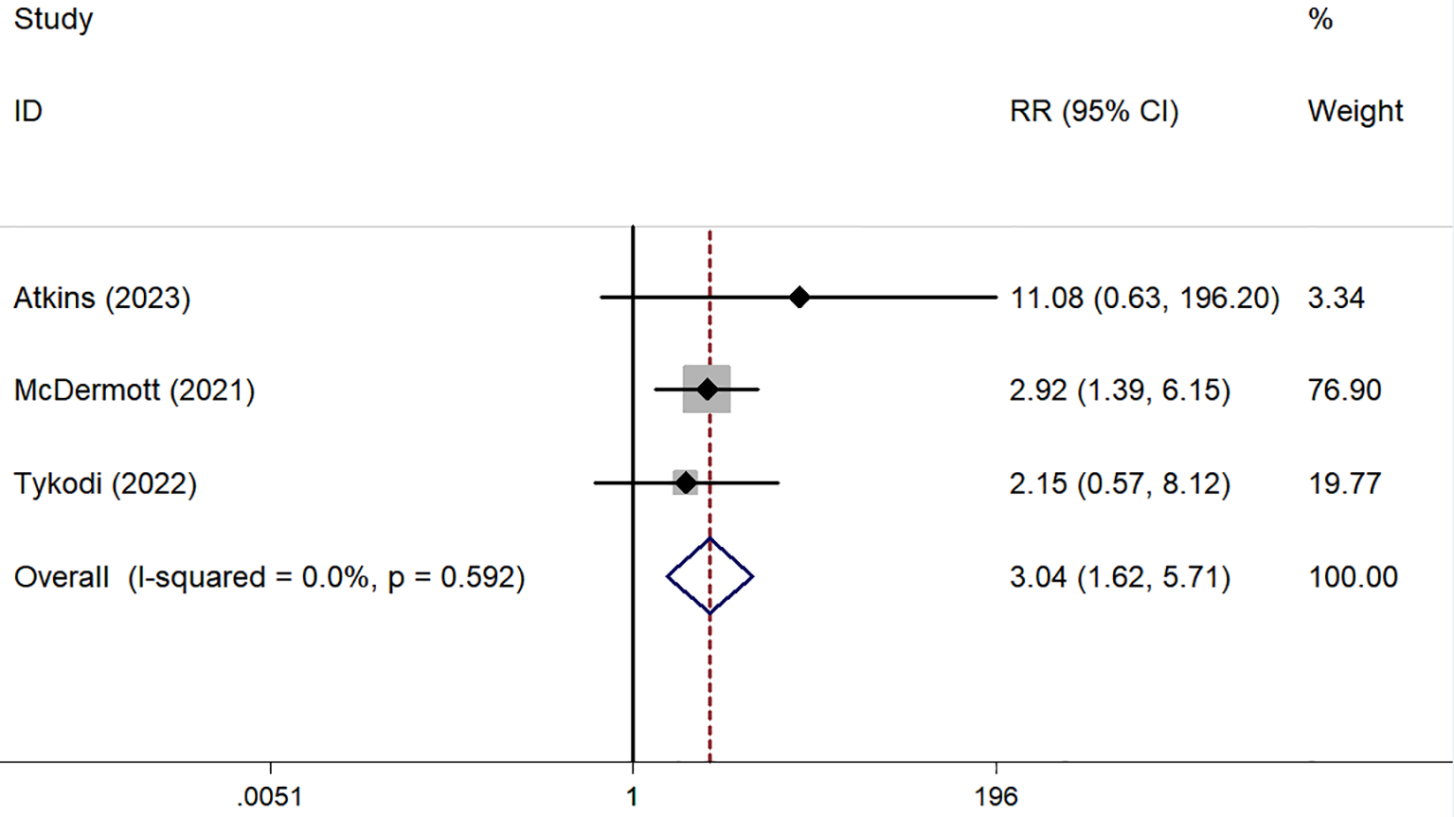
The forest plot comparing of ORR between PD-L1 positive group and PD-L1 negative group.

ORR was reported in six prospective (45–50) and five retrospective studies (51–55), while DCR was reported in five prospective (45–48, 50) and five retrospective studies (51–55). Both ORR and DCR showed no significant heterogeneity. The overall pooled ORR was 16% (95% CI: 13–19%) (**Figure 4C**). Subgroup analysis showed a pooled ORR of 18% (95% CI: 14–22%) in the prospective group and 13% (95% CI: 8–18%) in the retrospective group. The overall pooled DCR was 54% (95% CI: 50–58%), with a DCR of 58% (95% CI: 53–63%) in the prospective group and 46% (95% CI: 38–53%) in the retrospective group (**Figure 5C**). Additionally, ORR (RR = 3.044; 95% CI: 1.623-5.709%; P = 0.001) was higher in the PD-L1 positive group compared to the PD-L1 negative group (**Figure 6**).

### 3.8 Single-arm trials with chemotherapy

#### 3.8.1 Characteristics

A total of 142 patients participated in four phase II trials (56–59) and one retrospective study (60), all excluding prior chemotherapy. Three patients received first-line treatment (56, 59, 60). Tsimafeyeu (58) focused on capecitabine monotherapy, while the other studies used combination chemotherapy. Oudard (56) and Rizzo (60) exclusively studied patients with CDC, whereas the remaining studies included various types of nccRCC. Detailed information is presented in **Table 3** and **Table 4**.

#### 3.8.2 ORR and DCR

Both ORR and DCR showed significant heterogeneity, with an I² > 50% in the Q-test. ORR ranged from 0% to 26.09% across studies, resulting in a pooled ORR of 16% (95% CI: 6–28%) (**Figure 4D**). DCR, which was not reported in Richey (57), ranged from 47.22% to 72.55%, with a pooled DCR of 62% (95% CI: 48–75%) (**Figure 5D**).

### 3.9 Single-arm trials with combination therapy

#### 3.9.1 Characteristics

This analysis included ten studies with a total of 523 patients (61–70). Of these, one study incorporated both prospective and retrospective data (66), while the others were prospective phase II trials (61–65, 67–70). All studies investigated anti-angiogenesis therapies, using either small molecule targeted agents, such as VEGFR-TKIs, or large molecule monoclonal antibodies, such as bevacizumab, often in combination with mTORi, ICIs, or chemotherapy. Notably, Suarez (69) focused exclusively on pRCC, and Sheng (70) examined only CDC. The remaining studies included various subtypes of nccRCC, with pRCC being the most common subtype. Detailed information on these ten single-arm studies is presented in **Table 3** and **Table 4**.

#### 3.9.2 ORR and DCR

All studies provided data on ORR (61–70), while only eight included DCR (61–64, 67–70). Statistical analysis indicated high heterogeneity for both ORR (I² = 76.59%, P < 0.01) and DCR (I² = 89.75%, P < 0.01). ORR across studies ranged from 7.69% to 50.6%, with a pooled estimate of 36% (95% CI: 27–44%) (**Figure 4E**). DCR varied from 36.59% to 97.50%, resulting in a pooled DCR of 81% (95% CI: 70–91%) (**Figure 5E**). Subgroup analysis revealed five studies using bevacizumab (62–66), seven utilizing VEGFR-TKIs (61, 63, 66, 68, 70), and five based on ICIs (63, 65, 67–69). All subgroups demonstrated significant heterogeneity for ORR. The pooled ORRs were 31% (95% CI: 17–45%) for bevacizumab, 41% (95% CI: 31–50%) for VEGFR-TKIs, and 40% (95% CI: 31–50%) for ICIs.

## 4 Discussion

The optimal therapeutic strategy for nccRCC remains contentious. The heterogeneity of nccRCC complicates the establishment of robust evidence for specific therapies, as it hinders the conduct of prospective randomized trials. While systemic treatments effective for ccRCC show some activity in nccRCC, their response rates are significantly lower. Therefore, the panel recommends prioritizing clinical trial enrollment for nccRCC patients (1).

Our analysis of six RCTs compared the efficacy of mTORi and VEGFR-TKIs. Unfortunately, we did not achieve positive results in terms of effectiveness and survival. However, subgroup analysis of the four first-line studies showed that PFS was superior with sunitinib compared to mTORi (RR = 1.387; 95% CI: 1.04-1.85; p = 0.026). This advantage was not reflected in ORR and DCR, possibly due to limited data, as only three articles reported on ORR and DCR. Further validation through large-scale studies is needed.

NccRCC is a heterogeneous disease with significant variability among studies in histological subtypes, populations recruited, and risk factors, complicating comparisons across studies. Despite these challenges, current research suggests sunitinib as a potential treatment option for advanced nccRCC. The ESPN study found that sunitinib tended to prolong OS in first-line treatment of nccRCC without sarcomatoid features (15). Among 49 patients without sarcomatoid features, median OS was 31.6 months with sunitinib compared to 10.5 months with everolimus (p = 0.075) (15). The ASPEN trial highlighted the benefit of VEGFR-TKI therapy in patients with good or intermediate risk according to MSKCC criteria compared to everolimus (16). The RECORD-3 study, through prespecified subgroup analysis of MSKCC prognosis, found that median PFS was longer for patients with favorable and intermediate risk treated with first-line sunitinib compared to everolimus (18). However, this study included both ccRCC and nccRCC, and did not separately report results for the nccRCC group based on MSKCC criteria. These findings indicate that future nccRCC studies should focus on differences in efficacy based on MSKCC risk groups.

Sunitinib is a primary treatment option for nccRCC, but the search for a superior tyrosine kinase inhibitor (TKI) continues. Cabozantinib, an oral inhibitor of MET, VEGFR, and AXL, has shown promise. The randomized phase II SWOG 1500 trial (71) compared cabozantinib, crizotinib, and savolitinib with sunitinib in patients with advanced pRCC who had received up to one prior systemic therapy, excluding VEGFR- and MET-targeted TKIs. Only cabozantinib demonstrated significantly longer PFS and a higher ORR than sunitinib. As a dual VEGF-MET inhibitor, cabozantinib’s efficacy suggests that pRCC may involve both VEGF and MET signaling pathways. Although the SWOG 1500 trial focused on pRCC, the Meet-URO 2/NCT03354884 (20) trial treated 23 patients with CDC using cabozantinib, achieving an ORR of 34.78%, surpassing most previously reported targeted therapies. Consequently, NCCN guidelines now recommend cabozantinib as a preferred option alongside clinical trials (1).

Future research should focus on the genetic and molecular characteristics of nccRCC to better identify the target audience for these therapies. Given the limited number of RCTs, our analysis includes relevant single-arm trials to evaluate drug efficacy more comprehensively. Single-arm trials have shown limited efficacy of monotherapy for nccRCC, with mTORi demonstrating the lowest pooled ORR of only 6% (95% CI: 0-16%). In contrast, VEGFR-TKIs, chemotherapy, and ICIs had monotherapy effective rates of 14-16%. Koh (43) found that patients with chRCC treated with everolimus exhibited longer PFS and better ORR compared to other RCC subtypes. Conversely, Lee (44) reported no significant differences in PFS or OS among histological subtypes treated with temsirolimus, although patients with poor prognosis, as defined by ARCC criteria, had significantly shorter PFS and OS.

The pooled ORR for single-drug treatments with chemotherapy, VEGFR TKIs, and ICIs ranged from 14-16%. While the ORR and DCR of chemotherapy were similar to those of VEGFR-TKIs and ICIs, four of the five chemotherapy-related studies were published between 2007 and 2013, making them somewhat outdated. Additionally, the chemotherapy regimens varied: Tsimafeyeu (58) used oral capecitabine as a single agent for nccRCC, Richey (57) conducted a phase II trial with pemetrexed and gemcitabine, and the other three studies employed platinum-based combination chemotherapy (56, 59, 60).

Retrospective and recent prospective clinical trials have evaluated the antitumor activity of ICIs for nccRCC, either as monotherapy or combined with PD-1/PD-L1 inhibitors. Subgroup analysis revealed that PD-L1 positive patients had significantly better ORRs than PD-L1 negative patients, suggesting that PD-L1 expression levels could guide treatment choices for nccRCC patients. However, only three trials provided relevant data, and there was no standardized method for PD-L1 detection (46–48). Larger and more rigorous studies are needed to identify nccRCC patients who would benefit most from ICIs.

Treatment options for nccRCC are expanding, with drug combinations of different mechanisms entering clinical practice. Examples include lenvatinib plus everolimus, everolimus plus bevacizumab, pembrolizumab plus lenvatinib, and cabozantinib plus nivolumab. Our research indicates that combination therapies with different mechanisms show better ORR and DCR compared to single-agent therapies. Subgroup analysis found that combination therapies based on VEGFR-TKIs and ICIs achieved similar ORRs, both outperforming combination therapies based on bevacizumab.

## 5 Conclusions

In conclusion, the systematic review shows that sunitinib provides superior PFS compared to mTORi as a first-line treatment for advanced nccRCC. Due to limited data, single-arm trials were included to improve clinical guidance. The results indicated that PD-L1 positive patients had better ORR than PD-L1 negative patients, suggesting a need for further investigation. Additionally, combination therapies involving different mechanisms, especially those based on VEGFR-TKIs or ICIs, were more effective than single-agent treatments.

## Data Availability

The original contributions presented in the study are included in the article/supplementary material. Further inquiries can be directed to the corresponding author.
